# Serious game as an educational tool to promote the health of children and adolescents with cancer

**DOI:** 10.17533/udea.iee.v43n1e02

**Published:** 2025-04-14

**Authors:** Abigail Victória de Sousa Biró, Juliana Andreia de Souza Fernandes, Sheila Milena Pessoa dos Santos Fernandes, Luana Carla Santana Ribeiro, Giseli Cassiano de Almeida, Gabriele Cassiano de Almeida

**Affiliations:** 1 Nurse. E-mail: abigail.victoria@estudante.ufcg.edu.br. Corresponding author. https://orcid.org/0009-0007-6517-0376 Universidade Federal de Campina Grande Brazil abigail.victoria@estudante.ufcg.edu.br; 2 Nurse, Ph.D. Assistant Professor. Email: juliana.andreia@professor.ufcg.edu.br https://orcid.org/0000-0002-2990-7744 Universidade Federal de Campina Grande Brazil juliana.andreia@professor.ufcg.edu.br; 3 Nurse, Ph.D. Assistant Professor. Email: sheila.milena@gmail.com https://orcid.org/0000-0001-9396-9192 Universidade Federal de Campina Grande Brazil sheila.milena@gmail.com; 4 Nurse, Ph.D. Assistant Professor. Email: luana.carla@professor.ufcg.edu.br https://orcid.org/0000-0003-3485-3100 Universidade Federal de Campina Grande Brazil luana.carla@professor.ufcg.edu.br; 5 Ux Designer, Master student. Email: alcass.giseli@gmail.com https://orcid.org/0009-0001-3209-0747 Universidade Federal de Campina Grande Brazil alcass.giseli@gmail.com; 6 Nurse. Email: gabi.cassiano123@gmail.com https://orcid.org/0000-0001-6672-7817 Universidade Federal de Campina Grande Brazil gabi.cassiano123@gmail.com; 7 School of Nursing, Federal University of Campina Grande, Campina Grande, Paraíba, Brazil. Universidade Federal de Campina Grande School of Nursing Federal University of Campina Grande Campina Grande Paraíba Brazil; 8 Graduate in Design, Federal University of Campina Grande, Campina Grande, Paraíba, Brazil. Universidade Federal de Campina Grande Graduate in Design Federal University of Campina Grande Campina Grande Paraíba Brazil

**Keywords:** games and toys, educational technology, child, adolescent, neoplasms, health promotion., jogos e brinquedos, tecnologia educacional, criança, adolescente, neoplasias, promoção da saúde, juegos y juguetes, tecnología educativa, niño, adolescente, neoplasias, promoción de la salud

## Abstract

**Objective.:**

To develop and evaluate the usability of a serious game as an educational tool for promoting the health of children and adolescents with cancer.

**Methods.:**

The Contextualized Instructional Design (CID) methodology was applied to develop the serious game, structured in five stages: analysis, design, development, implementation, and evaluation. Usability assessment included children and adolescents diagnosed with cancer in a public federal referral hospital in a city in Northeastern Brazil, from April to June 2024. A sociodemographic questionnaire and the System Usability Scale with values ​​ranging from 0 to 100 points were used.

**Results.:**

The serious game, entitled Oncoped: on the health journey, is an educational and playful board game that contains eight personalized Paper Toys, 35 houses, and 100 cards divided into multiple-choice questions about cancers, diagnosis, treatments, and challenges; and cards containing information and care tips. The usability assessment was carried out by 12 hospitalized children and 3 adolescents, who after using the game filled out the information on the scale, resulting in a mean score of 95.16 points.

**Conclusion.:**

*The serious game Oncoped: on the health journey* is a fun and playful educational tool that provides effective and active learning. The educational technology received an excellent usability assessment among the game's target audience. Thus, it was found that the tool is innovative and has good acceptability for teaching and promoting the health of children and adolescents with cancer.

## Resumo

**Objetivo.:**

Desenvolver e avaliar a usabilidade de um *serious game* como ferramenta educativa para a promoção da saúde de crianças e adolescentes com câncer.

**Métodos.:**

Para a construção do *serious game*, foi aplicada a metodologia do Design Instrucional Contextualizado (DIC), estruturada em cinco etapas: análise, design, desenvolvimento, implementação e avaliação. Para a avaliação da usabilidade, incluiu crianças e adolescentes diagnosticados com câncer, em um hospital público federal de referência de uma cidade do Nordeste do Brasil, no período de abril a junho de 2024. Utilizou-se um questionário sociodemográfico e o System Usability Scale com valores de 0 a 100 pontos.

**Resultados.:**

O *serious game* intitulado Oncoped: na jornada da saúde, é um jogo de tabuleiro educativo e lúdico, que contém oito *Paper Toys* personalizados, 35 casas e 100 cartas divididas em perguntas de múltiplas escolhas sobre cânceres, diagnóstico, tratamentos e desafios; e cartas contendo informações e dicas de cuidados. A avaliação de usabilidade foi realizada por 12 crianças e 3 adolescentes hospitalizados, que após utilização do jogo preencheram as informações da escala que resultaram no score médio de 95.16 pontos.

**Conclusão.:**

O *serious game Oncoped: na jornada da saúde* é uma ferramenta de educação lúdica e divertida, que proporciona o aprendizado de forma efetiva e ativa. A tecnologia educativa obteve ótima avaliação de usabilidade entre o público-alvo do jogo. Assim constatou-se a ferramenta é inovadora e possui boa aceitabilidade para o ensino e promoção à saúde de crianças e adolescentes com câncer.

## Resumen

**Objetivo.:**

Desarrollar y evaluar la usabilidad de un juego serio como herramienta educativa para promover la salud de niños y adolescentes con cáncer.

**Métodos.:**

Para construir el serious game se aplicó la metodología de Diseño Instruccional Contextualizado (DIC), estructurada en cinco etapas: análisis, diseño, desarrollo, implementación y evaluación. Para la evaluación de usabilidad se incluyeron niños y adolescentes diagnosticados con cáncer en un hospital público federal de referencia en una ciudad del Nordeste de Brasil, de abril a junio de 2024. Se utilizó un cuestionario sociodemográfico y la Escala de Usabilidad del Sistema con valores de 0 a 100 puntos.

**Resultados.:**

El serious game titulado Oncoped: en el viaje de la salud, es un juego de mesa educativo y lúdico, que contiene ocho Paper Toys personalizados, 35 casas y 100 tarjetas divididas en preguntas de opción múltiple sobre cánceres, diagnóstico, tratamientos y desafíos; y cartas con información y consejos de cuidado. La evaluación de usabilidad fue realizada por 12 niños y 3 adolescentes hospitalizados, quienes luego de utilizar el juego llenaron la información de la escala que arrojó un puntaje promedio de 95.16 puntos.

**Conclusión.:**

*El* serious game *Oncoped: en el viaje de la salud* es una herramienta educativa divertida y lúdica que proporciona aprendizaje de una manera efectiva y activa. La tecnología educativa recibió una excelente calificación de usabilidad entre el público objetivo del juego. De esta forma, se encontró que la herramienta es innovadora y tiene buena aceptabilidad para la enseñanza y promoción de la salud de niños y adolescentes con cáncer.

## Introduction

Cancer is a disease characterized by the uncontrolled growth and division of cells, and can present different manifestations and causes. Tumors that affect children and adolescents generally grow faster and are more invasive. They are predominantly of embryonic origin, unlike those that affect adults, which are mostly related to exposure factors and lifestyle^1^ According to the National Cancer Institute, childhood cancer in Brazil is the leading cause of death (8% of the total) due to the disease in children and adolescents aged 1 to 19 years.^2^ It is estimated that, on mean, 29,000 children under the age of 9 will develop cancer annually in Latin America.^3^ However, progress in the treatment of childhood and adolescent cancer over the last four decades has proven to be quite effective, since, after treatment, 80% of patients respond with a cure, when early diagnosis occurs and appropriate treatment is carried out in specialized centers.^2^

However, treatment raises numerous doubts for children and adolescents, especially in the therapeutic process, due to the social, physiological and psychological changes in childhood. Furthermore, it is a situation that generates anxiety, especially when it involves invasive and painful procedures, such as chemotherapy and radiotherapy treatments.^4^ During this stage of care, it is essential that there is access to information, so that this public understands the process and, thus, can identify and prevent future discomfort or complications that may arise as a result of the treatment.^5^ To help in the understanding of the health-disease process, technological tools have been used, which can contribute to improving care, education, motivation and autonomy. Thus, innovative strategies can encourage changes in lifestyle habits and generate learning from these technologies.^6^ The use of educational tools with the objective of promoting health and providing guidance on childhood and adolescent cancers, their treatments, diagnoses and interventions helps to clarify doubts, fill knowledge gaps, induce behavior changes and stimulate decision-making.^7^

Serious games are technological games developed for educational purposes, and not just for entertainment. This technology uses a methodology that enables learning about the disease, so that this knowledge occurs through the dynamics of the game and interaction between participants, in addition to being a playful and fun way. Thus, the patients can enjoy a common tool in their daily lives, such as games, to acquire knowledge about the new situation that was previously unknown.^8^ In the health area, several studies have highlighted the importance of using serious games for health care for children and adolescents, such as “Hospital Mirim”, which aims to cope with pain and the context of the invasive blood collection procedure in children,^9^ and “FonoConnect”, which aims to be a playful tool for patients, in order to make speech therapy a pleasant and stimulating moment.^10^ However, these serious games were developed virtually, and no serious games were found for the purpose of educating children and adolescents with cancer, which demonstrates the relevance of this study.

In this context, the educational process regarding the disease and the treatment process is extremely important to prevent discomfort and complications resulting from this phase of care. To this end, educational games are extremely important as a playful means of offering relevant information through play. Thus, this study aimed to develop and evaluate the usability of a serious game as an educational tool for promoting the health of children and adolescents with cancer. 

## Methods

This is a methodological research, in the form of technological production, which aims to build an educational serious game to promote the health of children and adolescents with cancer. The research was developed from October 2023 to August 2024. To build the serious game, the Contextualized Instructional Design (CID) methodology^11^ was applied, which consists of the intentional action of planning, developing and applying specific teaching situations. CID is carried out from five distinct stages, namely: analysis, design, development, implementation and evaluation.

The analysis stage consisted of identifying learning needs, defining instructional objectives and identifying the restrictions involved.^11^ To this end, a scoping review was carried out, subdivided into five stages: identification of the research question; identification of relevant studies; selection of studies; systematization of data; and collection, cataloging and presentation of results, through which the information that makes up the theoretical framework of this serious game was obtained. In the review, searches were conducted in the Virtual Health Library (VHL) and the U.S. National Library of Medicine of the National Institute of Health (PubMed), using the Health Sciences Descriptors (DeCS) and the Medical Subject Heading Terms (MeSH): “child”, “adolescent”, “neoplasms”, “chemotherapy”, “surgery”, “radiotherapy” and “signs and symptoms”. In addition to the theoretical basis outlined through the scoping review, the content of the serious game was also composed of information present in the manuals of the José Alencar Gomes da Silva National Cancer Institute (INCA). This content was used to create the serious game and was validated by a group of experts in the health field, who had knowledge about pediatric oncology and/or technological development, through analysis of the Content Validation Index (CVI), which measures the proportion of agreement of the research participants, for each item analyzed and on a general scale.^12^

In the design stage, the layout was created with the collaboration of a professional UI/UX Designer, with the help of Adobe Illustrator software, whose main function is to work with vector illustrations, which in this research was used to create the entire graphic part and visual identity of the game, from the logo, the board, to the cards and pins. Once this stage was completed, the third stage began, which included the development of the educational didactic content, the questions and answers on the cards, as well as the rules of the serious game. 

In the implementation stage, the design proposal was adapted to the game, and for this purpose a printed board was created on paper, with pins, question and answer cards, along with some additional information about the cancer treatment process, challenge cards and cards with health care tips. After the serious game was created, a usability assessment was carried out, which is equivalent to the monitoring, review and maintenance of the proposed tool; this is the moment when the target audience can influence the construction and improvement of the serious game, carried out based on responses from the application of the “System Usability Scale” (SUS), with values ​​ranging from 0 to 100 points.

The usability assessment was carried out with children and adolescents with cancer, with the number of participants in the sample following the recommendation of Pasquali,^13^ which establishes a minimum number of 6 and a maximum of 20 individuals, which resulted in the final sample with 15 individuals. Data were collected at a federal public referral hospital that treats children and adolescents with cancer in the city of Campina Grande, Paraíba, Brazil, from April to June 2024. The inclusion criteria were literate children and adolescents diagnosed with cancer and undergoing antineoplastic treatment. The exclusion and discontinuation criteria were children with low cognitive levels who did not understand the rules of the game or who were unable to continue playing the game due to complications resulting from the side effects of the treatment or the disease itself. The children and adolescents were invited to participate after the objectives and procedures of the research were explained. Upon acceptance of the invitation, the Informed Consent Form (ICF) was completed by their guardians and the Informed Assent Form (AAS) was completed by minors.

A sociodemographic questionnaire was applied to characterize the participants, which contained the following variables: sex, age, diagnosis, and treatment time. Participants were instructed to play the serious game and respond to the usability assessment scale “System Usability Scale” (SUS) in its version validated for the Portuguese language by Tenório.^14^ For each topic, the ten items were analyzed and assigned a value, each of which has a Likert-type scale, ranging from: 1 = strongly disagree; 2 = partially disagree; 3 = neither agree nor disagree; 4 = partially agree; and 5 = strongly agree. The even-numbered topics 2, 4, 6, 8, and 10 correspond to negative responses; their scores are equivalent to subtracting five minus the number given on the Likert scale; while the odd-numbered topics 1, 3, 5, 7, and 9 correspond to positive responses; their scores are equivalent to subtracting one from the number on the Likert scale. After quantifying the values ​​obtained in each topic, they were added and multiplied by 2.5; the results obtained ranged from 0 to 100 points, obtained on the System Usability Scale^15^. Furthermore, the measurements made by the sample participants were evaluated in a relevant manner, in order to contribute to improvements in the tool, content or functionality of the serious game. The serious game was considered satisfactory, sufficient, attractive and relevant, from the point of view of usability, if it achieved scores that were judged as the best performance in the instrument applied by the SUS, with a mean score between 70 and 100.^15^

This research followed the recommendations of Resolution 466/12 of the National Health Council, which regulates the conduct of research involving human beings, and was approved by the Research Ethics Committee of the Federal University of Campina Grande, under Opinion number 5,654,700 and CAAE number 61249622.4.00005182. 

## Results

**Figure 1:**
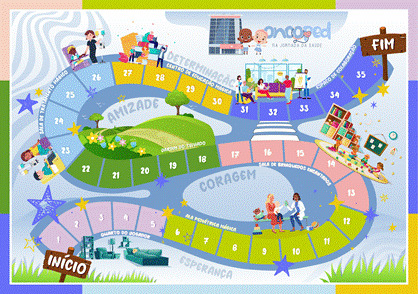
Board model

Note: Início = Start; Esperança = Hope; Coragem = Courage; Amizade = Friendship; Determinação = Determination; Fim = End

The present study resulted in the development of a serious game called Oncoped: on the health journey, composed by combining the terms oncology and pediatrics, which alludes to the target audience, which are children and adolescents with cancer, and the health journey, which reflects the situation they are exposed to due to the disease and the treatment in search of a cure. The serious game Oncoped: on the health journey has as its main theme the experience of the target audience diagnosed with cancer, undergoing antineoplastic treatment. The layout of the serious game and the structure of the board and cards were defined with drawings, images and coloring experienced by children and adolescents in the hospital environment, such as the treatment room and the playroom. The aesthetics used with soft but diverse colors aimed to attract the attention of the public (Figure 1). 

**Figure 2:**
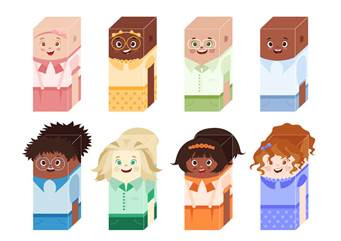
Paper toys

The board was developed with 35 squares and 100 cards. The game content contains educational multiple-choice questions in accessible language about cancers, antineoplastic treatment, challenges faced by children with cancer, diagnosis, signs and symptoms resulting from treatment and the most common side effects. In addition, it has cards with information about the importance of doing physical exercise that fits into one's routine, challenges to be carried out with other players, health care tips, curiosities related to cancer and personal care guidelines. Paper Toys (figure 2) were used as pegs, which are personalized markers for each player on the board, which resemble the participants. To this end, the different races and genders were respected, as well as some characters with no hair, because one of the side effects of cancer treatment is hair loss, which can be partial or total (NIC, 2018) and others with hair, because there are drugs that do not cause this effect. A total of eight Paper Toys were created. 

The serious game board was printed on two A3 sheets, measuring 42 cm in height and 59.4 cm in width. The cards are on polaseal paper, measuring 9 cm in height and 6 cm in width; the instruction manual was printed on A4 sheets, measuring 21 cm in width and 29.7 cm in height; and the Paper Toys were produced in a material combining polyethylene chloride (PVC) and polyethylene terephthalate (PET), measuring 2 cm in width and 5 cm in height. The roll and move mechanism was used to develop the serious game mechanism, which consists of the player rolling the dice and moving the pins on the board according to the number reached on the dice; luck is the main factor in this mechanic. The point-to-point movement allows the player to occupy points on the board using the pins, connected by lines that serve as the basis for the movements and selection of cards. In this way, it will be possible to choose a card from a limited number available. The usability assessment of the serious game was carried out with 15 participants (Table 1). Most of the children and adolescents had incomplete elementary education (80%), had a monthly family income between one and two minimum wages (60%), used cell phones (93.3%), and played board games (66.67%). The predominant type of cancer was leukemia (66.67%) and the main treatment was chemotherapy or chemotherapy combined with radiotherapy (46.67%).

**Table 1:** Profile of the 15 hospitalized children and adolescents with cancer

Variable	N	%
Gender		
Female	6	40
Male	9	60
Age		
2 to 4 years	4	26.67
5 to 7 years	4	26.67
8 to 10 years	4	26.67
11 to 18 years	3	20
Education		
Illiterate	3	20
Incomplete Elementary School	12	80
Family Income		
< 1 minimum wage	3	20
1 to 2 minimum wages	9	60
2 to 3 minimum wages	3	20
Number of people living in the house		
2 to 3	5	33.33
4	7	46.67
5	3	20
Race		
White	7	46.67
Mixed-race	8	53.33
Uses cell phone		
Yes	14	93.33
No	1	6.67
Mean hours using cell phone		
1 to 2	7	46.67
3 to 4	4	26.67
5 to 6	2	13.33
7 to 8	2	13.33
Plays board games		
Yes	10	66.67
No	5	33.33
Has a board game at home		
Yes	6	40
No	9	60
How often do you play?		
Rarely	8	53.33
Once a week	3	20
Once a month	4	26.67
Type of cancer		
Leukemia	10	66.67
Lymphoma	1	6.67
Others	4	26.67
Type of treatment		
Chemotherapy	7	46.67
Chemotherapy + Radiotherapy	7	46.67
Surgery	1	6.67

After the children or adolescents played, they answered the SUS questionnaire (Table 2). The following items were evaluated: ease of play, autonomy, variety of content and age appropriateness. The scores for each participant in the sample obtained through statistical analysis were: mean of 95.16, mode of 100 and median of 97.5. Thus, the usability evaluation of this serious game was classified as “acceptable”, considering that its mean SUS score is 95.16. 

**Table 2:** System Usability Scale (SUS) scores of the 15 survey participants

Participant	SUS Score
1	77.5
2	87.5
3	90
4	90
5	95
6	100
7	100
8	95
9	97.5
10	100
11	97.5
12	100
13	100
14	97.5
15	100
SUS mean score	95.16

## Discussion

In the last decade, technological advances in the health field have increased the relevance of research on this topic aimed at improving patients' quality of life. From this perspective, technological tools, such as serious games, represent one of the various groups of technologies used for leisure and interaction, which are common in the daily lives of children and adolescents. These tools can also be applied as educational strategies to promote better learning and memorization of certain subjects, as they allow for better engagement and improve health information and therapeutic communication.^7^

Currently, in Brazil, there are websites and manuals on cancer for children and adolescents of an informative and educational nature^2^^,^^16^ that allow users to be aware and serve as support for frequently asked questions. However, no serious games of an educational nature were found aimed at the use of hospitalized patients in the established age group. Thus, Oncoped: on the health journey, is considered an innovative technology for teaching and nursing care provided to this public, capable of helping to clarify doubts and provide new information based on scientific evidence in an accessible manner and promoting quality in care for children and adolescents with cancer. The use of the mechanisms used in the serious game through the game cards, which contain questions and information, is also shown in another study carried out in the United States, which evaluated, through focus groups, with adolescents aged 12 to 14, the learning about cancer prevention through serious games.^18^ In the study, the participants reported the importance of using this methodology for learning, especially because it is a fun and active medium, which makes the educational action more effective and improves the chances of retaining the subject, unlike a lecture or reading a text. In addition, this type of technology can promote encouragement for changing behaviors and making daily decisions.

Such technology can facilitate nursing care by providing information and disseminating knowledge about health promotion for children and adolescents.^19^ Furthermore, the use of narratives and illustrations that are found in the daily lives of children and adolescents are shown to be technological strategies that describe the content and facilitate learning, in addition to encouraging decision-making and making them analyze daily care and self-care behaviors.^20^ The need to use illustrations that resemble the experiences encountered and with which they identify is a way to ensure learning in a playful and fun way.^21^ Thus, the layout chosen for Oncoped: on the health journey has attractive and fun elements close to the reality experienced, with the purpose of arousing the interest of children and adolescents in the subject and contributing to improving the care provided to this audience. 

The behavior of participants during the use of Oncoped: on the health journey corroborates the understanding that the device met the purpose of being attractive. It was observed that the children and adolescents participating in the research were happy and excited during the application of the game, with time for fun, sharing experiences and knowledge. In this regard, another study highlights the importance of children and adolescents participating in analog games such as board games and cards, as they promote moments of reflection and leisure through experience, in order to expand knowledge and use imagination.^22^ Regarding the usability assessment, an essential stage for the knowledge and improvement of the proposed product, the mean was 95.16. It was considered that the desired mean was reached, since the literature indicates a mean greater than or equal to 91 points as adequate.^15^

Among the implications for clinical practice, it is worth noting that Oncoped: on the health journey promotes moments of relaxation and interaction, through play, for children and adolescents undergoing antineoplastic treatment. Furthermore, it allows nursing professionals to share information relevant to health promotion and coping with the disease and treatment. For future studies, it is recommended that professionals be trained to use serious games in their routine as a means of disseminating knowledge, since they are responsible for enhancing learning.

A limitation of this study is the difficulty in finding low-cost materials for producing the serious game that would meet the need for it to be sanitizable for use in the hospital.

This study enabled the development of a serious game as an educational tool for health promotion for children and adolescents with cancer. According to the usability assessment, the game was considered adequate. It is considered that the technology developed can contribute significantly to the dissemination of health and nursing information and knowledge on the subject, to interactions between professionals, children and adolescents, in addition to providing distraction, fun and joy during the game. Furthermore, the game was well-received by the target audience, as patients identified with Paper Toys that were similar to them and found information about situations they had experienced in the cards.

Therefore, it is seen as a contribution to the hospital context that serious games can contribute to the excellence of the quality of care provided by nursing professionals and to the construction of bonds with children and adolescents with cancer, as a means of communication and rapport. The aim is for serious games to be introduced as an educational tool in health services, as they are an innovative educational technology that is easy to apply, well-accepted and capable of disseminating important information about the subject to patients undergoing cancer treatment. 
